# The bile acid-CoA ligase, FATP5, is necessary for the synthesis of N-acyl taurines in the liver

**DOI:** 10.1016/j.jlr.2026.101012

**Published:** 2026-03-02

**Authors:** Katharina B. Kuentzel, Samuel A.J. Trammell, Anna S. Hassing, Benjamin P. Garfinkel, Ivan Bradić, Kathleen Tchoukoua, Matthew P. Gillum, Martin R. Larsen, Trisha J. Grevengoed

**Affiliations:** 1 Department of Biomedical Sciences, University of Copenhagen, Copenhagen, Denmark; 2 Alnylam Pharmaceuticals, MA, USA; 3 Department of Biochemistry and Molecular Biology, University of Southern Denmark, Odense, Denmark

**Keywords:** NAT, SLC27A5, Liver, Lipids, Fatty Acid/Transport, Bile acids and salts/metabolism, Bile acids and salts/Biosynthesis

## Abstract

N-acyl taurines (NAT) are endogenous, bioactive conjugates of fatty acids and taurine with roles in carbohydrate and lipid metabolism. In the liver, NATs are synthesized by bile acid-CoA:amino acid N-acyltransferase (BAAT), which also conjugates bile acids to taurine or glycine, suggesting an overlapping hepatic synthesis pathway. BAAT catalyzes the transfer of an acyl-chain from an activated coenzyme A (CoA) to taurine, but the hepatic enzyme responsible for synthesizing the acyl-CoA remains unknown. Using liver transcriptomics in mice unable to hydrolyze NATs, we identified *Slc27a5,* which encodes the acyl-CoA synthetase, fatty acid transport protein 5 (FATP5), as a potential regulator of hepatic NAT synthesis. In vivo knockdown of the enzyme confirmed that FATP5 is necessary for hepatic NAT synthesis and upstream of BAAT, likely through its acyl-CoA synthetase activity. The dual function of this enzyme in activating both fatty acids and bile acids for conjugation identifies a functional overlap between the hepatic NAT and bile acid production pathway.

## Graphical Abstract

**Figure:**
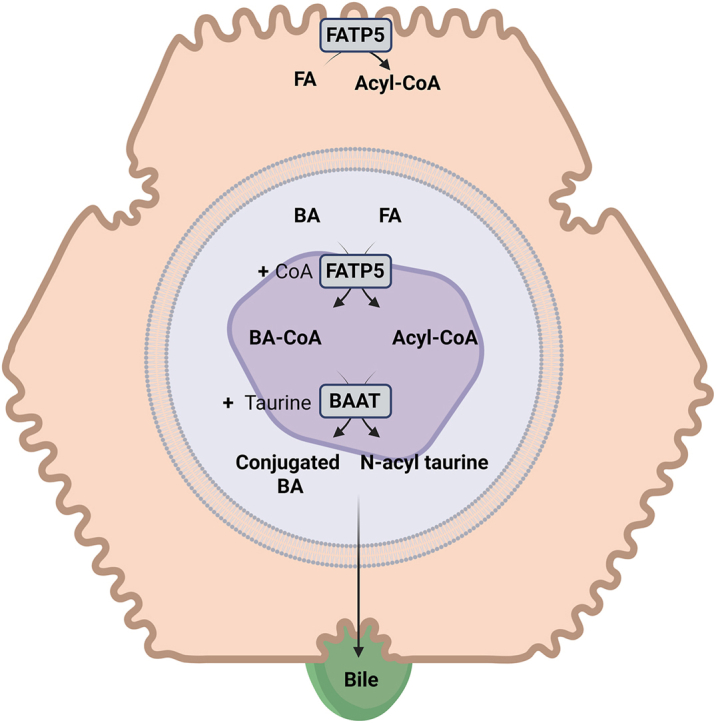

Bile acids are amphipathic cholesterol derivatives that emulsify dietary fat, allowing for their lipase-mediated breakdown and intestinal absorption. Additionally, bile acids function as signaling molecules, activating receptors, such as TGR5 and FXR, and thus serving as potent regulators of metabolism (1). To increase solubility and emulsifying properties of bile acids, bile acid-CoA:amino acid N-acyltransferase (BAAT) conjugates newly synthesized or recycled bile acids to taurine or glycine in hepatic peroxisomes before secretion into the gallbladder (2, 3, 4). Although conjugated bile acids represent a significant part of bile, other components may exert a similar function as detergents and/or signaling molecules. Besides well-studied and characterized bile acids, phospholipids, and cholesterol, another lipid family is abundant in bile: the N-acyl taurines (NATs).

NATs belong to a class of N-acyl amino acids and are lipid metabolites comprised of a fatty acyl-chain of variable length amidated to taurine. The amphipathic nature of the NAT molecules, containing a hydrophobic acyl chain and the hydrophilic taurine, increases the solubility of the fatty acid, similar to steroidal bile acid conjugates of taurine. NATs are evolutionarily conserved from aquatic animals (5, 6, 7) to mice (8, 9) and humans (10) and are found in various tissues and fluids, with the highest levels in bile, brain, plasma, and liver (8, 9, 10).

In contrast to bile acids, which are deconjugated by bacteria in the gut, NATs are hydrolyzed by the ubiquitously expressed fatty acid amide hydrolase (FAAH) (11). Pharmacological inhibition of FAAH results in a rapid, tissue-specific elevation of specific NATs. After FAAH inhibition, polyunsaturated fatty acid (PUFA)-containing NATs accumulate rapidly in the liver, plasma, and kidney, whereas long-chain saturated NATs accumulate upon prolonged FAAH blockage in the brain, spinal cord (8, 12), and skin (13). Thus, NAT abundance differs in the degree of saturation between the central nervous system and peripheral organs, and this difference does not depend upon FAAH, suggesting that synthetic enzymes drive the observed acyl-chain diversity.

The acyl-chain of the NAT appears to determine its biological activity. The oleic acid (C18:1)-containing NAT, oleoyl-taurine (OLE-T), is the most abundant NAT species in mouse and human plasma (9, 10) and is involved in the regulation of glucose metabolism. OLE-T induces GPR119-mediated GLP-1 secretion in mice to decrease food intake and improve glucose tolerance (9) and activates TRP ion channels (12). Long-chain saturated fatty acid-containing NATs regulate skin wound healing (13). PUFA-containing NATs are highly abundant in the liver (8) and bile (10), and can be increased by the supplementation of high-PUFA oils, such as fish oil, and impair intestinal fat absorption (10). This suggests that NAT abundance is regulated by substrate availability. Because the biological activity of NATs varies with the acyl chain and NAT abundance, identifying the NAT synthetic pathway is critical to understanding their role in biology.

Although the NAT degradation pathway has been studied over the past two decades (8, 9, 10, 11, 13, 14), little is known about the NAT synthesis pathway in vivo. Recently, NAT-synthesizing organs were identified, with the liver and kidney showing the highest NAT synthesis activity with a polyunsaturated acyl-CoA (12, 15). The hepatic enzyme BAAT—the bile acid-conjugating enzyme—has been identified as the hepatic PUFA-containing NAT synthase in humans and mice (15). With BAAT serving as both the bile acid-conjugating enzyme and NAT synthase, we hypothesized that these molecular classes overlap in the hepatic conjugation/synthesis pathway.

This study aimed to investigate the similarities between the hepatic NAT and bile acid synthesis pathways. Our experiments identified an unknown function of the liver-specific protein, fatty acid transport protein 5 (FATP5), in hepatic NAT production upstream of BAAT. This role in NAT synthesis was independent of FATP5's role in fatty acid uptake and retention or other metabolic regulation. Thus, we present an additional point of overlap between bile acids and NATs, which will be important in both understanding the roles of NATs as well as understanding the effects of altering bile acid metabolism through pharmaceuticals in development.

## Materials and Methods

### Animal care and approval

All mouse protocols were approved by the Danish Animal Experiments Inspectorate and performed according to Animal Research: Reporting of In Vivo Experiments (ARRIVE) guidelines. Mice were housed in a specific pathogen-free environment on a 12-h light/12-h dark cycle and maintained on a chow diet with ad libitum access to water and food unless otherwise specified. Male and female mice were included in the experiments to study whether the enzyme candidate is involved in NAT synthesis across both sexes, as specified in the corresponding studies. Female C57BL/6NTac mice were obtained from Taconic and acclimatized in the animal facility prior to siRNA-mediated knockdown experiments, and male WT mice were bred in-house from the FAAH S268D line. Male FAAH S268D mice and WT littermates on a C57BL/6N background (9) were fed either a high-fat diet (40% kcal from fat, 0.2% cholesterol, Research diets D22060302) for 3 days to perform RNA sequencing or a high-fat/sugar/cholesterol diet (HFFC, 40% kcal from fat, 20% kcal from fructose, 2% cholesterol, Research diets D09100310) for 4–5 weeks for the knockdown experiments. siRNA was injected subcutaneously (s.c.) and one of the following siRNAs (a kind gift from Alnylam): siBaat_1_, siBaat_2_, siFatp5, or vehicle (PBS) at a concentration of 2 or 10 mg/kg, was used. Animals were sacrificed two weeks after siRNA or vehicle injection unless otherwise specified, and tissues were collected in deep anesthesia and snap frozen.

### Blood and plasma collection

Blood was collected from the retroorbital plexus, considered systemic blood, into EDTA-containing tubes on ice, and centrifuged at 5,100 × g for 10 min at 4°C to obtain plasma. The plasma samples were stored immediately at −80°C until analysis. Plasma triacylglycerol (TR210 Randox), cholesterol (TC; CH200, Randox), nonesterified fatty acid (Fujifilm Wako Pure Chemical Corporation), and ALT (AL1205, Randox) measurements were performed with enzymatic kits according to the manufacturer's instructions.

### RNA isolation, cDNA synthesis, and real-time qPCR

Total RNA was extracted from pulverized liver using TRIzol (Thermo Fisher Scientific) according to the manufacturer's instructions. Then, 2 μg of RNA were reverse transcribed using High-Capacity cDNA Reverse Transcription Kit (Thermo Fisher Scientific), and 6 ng of complementary DNA (cDNA) were used for quantitative real-time PCR. Primer sequences can be found in Table 1. Gene expression was normalized to *Rpl13a1* and analyzed using the 2^−ΔΔCT^ method.

**Table 1:** Primer sequences used in real-time qPCR

Gene	Forward Sequence (5′-3′)	Reverse Sequence (5′-3′)
*Slc27a5*	GCATCCTTCGAATGCTGACTCC	CGAACCTTGGTCAAAAGAAGTCC
*Slc27a2*	TCGTGGAGGTCTGAAGTCACT	GATGGTTGCCGCTTTTGGAA
*Baat*	GGTTGCTGTAAAACTACTGTTTTGG	TGTGCACAGGCTCATCAACA
*Cyp7a1*	GGGAAGTTTCGACATGCTCTC	GTGGTTCTTGGAGGTGCCTT
*Tnfa*	GCTCCTCCACTTGGTGGTT	GTCTACTGAACTTCGGGGTGA
*Il1b*	AAATACCTGTGGCCTTGGGC	CTTGGGATCCACACTCTCCAG
*Cd14*	CTCTGTCCTTAAAGCGGCTTAC	GTTGCGGAGGTTCAAGATGTT
*Adgre1*	TTGTACGTGCAACTCAGGACT	GATCCCAGAGTGTTGATGCAA
*Mcp1*	TCTGGACCCATTCCTTCTTG	AGGTCCCTGTCATGCTTCTG
*aSma*	GTCCCAGACATCAGGGAGTAA	TCGGATACTTCAGCGTCAGGA
*Col1a1*	GCTCCTCTTAGGGGCCACT	CCACGTCTCACCATTGGGG
*Rpl13a1*	AGCCTACCAGAAAGTTTGCTTAC	GCTTCTTCTTCCGATAGTGCATC

### RNA sequencing

Hepatic RNA from male WT and FAAH S268D mice was extracted as described above and purified with the RNeasy kit (Qiagen). Total RNA sequencing libraries were prepared using the Illumina TruSeq Stranded total RNA Gold protocol (Illumina). The total RNA was depleted of rRNA by RiboZero beads, fragmented, and cDNA was synthesized using SuperScript III Reverse Transcriptase (Thermo Fisher Scientific). cDNA was adenylated to prime for adapter ligation and after a clean-up using AMPure beads (Beckman Coulter), DNA fragments were amplified using PCR followed by a final clean-up. Libraries were quality-controlled using a Bioanalyzer instrument (Agilent Technologies) and subjected to 51-bp paired-end sequencing on a NovaSeq 6,000 system (Illumina).

FASTQ-files were analyzed using the Subread (16) suite of software v1.6.4. Reads were aligned using the Subjunc tool against mm10 and the GENCODE (17) vM21 gene model using default parameters. Reads were summarized to genes using featureCounts (18) counting only uniquely mapped reads where both ends map, are overlapping a single feature, are mapped in a reverse-stranded manner, and where both end map to the same chromosome.

Testing for differential expression was performed using edgeR (19) v. 3.26.0. Raw counts were filtered to retain genes with sufficient expression across samples using the filterByExpr function. Normalization to account for differences in library sizes was performed using calcNormFactors. To estimate the dispersion of the data, the estimateDisp function was applied. Following normalization, gene counts were transformed into log counts per million for visualization purposes, including the generation of heatmaps and multi-dimensional scaling plots. For the identification of differentially expressed genes, a generalized linear model quasi-likelihood approach was used via the glmQLFit function, using a group means model of the form ∼ 0 + genotype + cohort. Contrasts were constructed so a positive logFC indicated an increase in the knock-in condition. The quasi-likelihood F-test was applied to determine differential expression between experimental conditions, ensuring robust control over false positives. Genes with a false discovery rate < 0.1 were considered significantly differentially expressed and used for the screening approach.

### Liver organelle preparation

Peroxisomes and other organelles were isolated via differential centrifugation. Briefly, mice were fasted overnight, and livers were homogenized in homogenization buffer (250 mM sucrose, 5 mM MOPS, 1 mM EDTA, 1 mM DTT with protease inhibitors (Sigma)). All subsequent steps were carried out at 4°C. Nuclei and unbroken cells were removed by centrifugation at 600 × g for 5 min, and heavy mitochondria were isolated from the resulting supernatant by centrifugation at 2,700 × g for 10 min. The light mitochondria and peroxisomes were spun down at 37,000 × g for 30 min. The resulting pellet was resuspended in 22.5% Optiprep (STEMCELL technologies incorporated, catalog # 07820). A gradient was then formed with 27.5% Optiprep, the mitochondria/peroxisome prep, and 20% Optiprep. The gradient was then centrifuged at 100,000 × g for 1.5 h, and peroxisomes were collected from the interface of 22.5% and 27.5% Optiprep layers. Plasma membrane-enriched fractions were collected from the top of the gradient. Purity of fractions was confirmed via Western blot.

### Western blot

Pulverized liver tissue was homogenized in RIPA buffer containing Halt™ Protease Inhibitor Cocktail (Thermo Fisher Scientific), and protein concentration was determined by Pierce™ Rapid Gold BCA protein assay (Thermo Fisher Scientific). Twenty micrograms of liver and 15 μg of organelle protein were separated on a 4%–12% SDS-PAGE and transferred to a polyvinylidene difluoride membrane. Primary antibodies against BAAT (PA596744, Thermo Fisher Scientific) and FATP5 (MA5-31908, Thermo Fisher Scientific) were incubated 1:2,000 in 5% nonfat milk-TBST at 4°C overnight. Control primary antibodies, GAPDH (ab9485, Abcam), catalase (ab209211, Abcam), and Na/K ATPase (ab76020, Abcam), were incubated at 1:20,000 using the same conditions. Horseradish peroxidase-conjugated secondary antibodies, rabbit anti-mouse (AB_2533933, Invitrogen) and goat anti-rabbit (ab6721, Abcam), were incubated for 2 h at room temperature prior to detection on a Bio-Rad ChemiDoc™ imaging system.

### Proteomics

Approximately 20 mg of pulverized liver tissue was lysed in 5% Sodium deoxycholate in 50 mM Hepes (pH 8.5) containing protease inhibitors (Roche Complete protease inhibitor EDTA free, 118735800001) by sonication for 30s at 60% amplitude. Afterward, proteins were denatured for 10 min at 90°C and centrifuged for 30 min at 20,000 × g twice to remove lipids. Protein concentrations were measured in the infranatant using a NanoDrop. Twenty micrograms of protein were reduced and alkylated with 10 mM DTT and 20 mM iodoacetamide, respectively. Proteins were digested with LysC 0.04 AU/mg protein for 30 min at 37°C, followed by the addition of 5% trypsin overnight at 37°C. The next day, proteins were digested further by the addition of 1% trypsin for one hour. Ten micrograms of proteins were transferred to a new tube, and sodium deoxycholate was precipitated with 2% trifluoroacetic acid. After centrifugation at 20,000 × g for 10 min, the supernatant was transferred to a C18 stage tip (Thermo Fisher Scientific) for peptide purification. Peptides were eluted with 65% acetonitrile in 0.1% trifluoroacetic acid, vacuum-dried, and resuspended in 0.1% formic acid.

### Mass spectrometry and data analysis

Approximately 800 ng of peptides, dissolved in solvent A (0.1% formic acid), were loaded onto a 150 mm long EASY-Spray™ HPLC Column (ES904) maintained at 50°C. The separation was performed using a Thermo Scientific™ Vanquish™ Neo UHPLC system coupled to an Orbitrap Astral mass spectrometer. Peptides were eluted from the column over a 40-min gradient by increasing the concentration of solvent B (95% acetonitrile and 0.1% formic acid) from 2% to 38%. The mass spectrometer was operated in positive ion mode. Full scan mass spectra (MS1) were acquired in the Orbitrap at a resolution of 240,000 with an automatic gain control target of 500%, a scan range of 400–1,000 m/z, and a maximum injection time of 5 ms. Data-independent acquisition was performed with a 2 m/z isolation window and higher-energy collisional dissociation set to 25%. The resulting fragment ions were detected in the Astral analyzer over a scan range of 150–2000 m/z with an automatic gain control target of 500% and a maximum injection time of 2.5 ms, using a total cycle time of 0.6 s.

Raw data files were searched with Spectronaut (20.1) using the *Mus musculus* reference proteome from UniProt (ID UP000000589_10090), containing 21,756 genes. The search was performed using the “BGS Factory Settings,”which included trypsin digestion with a maximum of two missed cleavages, carbamidomethyl (C) as a fixed modification, and up to five variable modifications, including protein N-terminal acetylation and oxidation (M). Identification was performed using the Deep DirectDIA + workflow. For quantification, precursor filtering was set to “Identified (Qvalue)” with no software-level imputation. The proteotypicity filter was set to “Only protein group specific,” and cross-run normalization was applied. Downstream bioinformatic analysis was performed in R (4.5.0) within a reproducible environment managed by the renv package. Protein group intensities were log_2_-transformed, and the dataset was filtered to retain proteins with valid values in at least 80% of the samples. Remaining missing values were imputed using the Quantile Regression-based Left-Censored method, as implemented in the DEP package. Differential protein abundance was determined using the limma package. A linear model with a group-means parameterization was fitted for each protein to accommodate the 2 × 2 factorial experimental design (genotype: WT/FAAH S268D; treatment: Vehicle/siFatp5). Specific contrasts were then defined to test for the main effects of genotype and treatment, as well as the statistical interaction between them. Empirical Bayes moderation was applied to the model to increase statistical power. Proteins with a Benjamini-Hochberg adjusted *P*-value < 0.05 and an absolute log_2_ fold change greater than a data-driven threshold were considered statistically significant. This fold-change threshold was determined independently for each contrast by calculating the 80th percentile of the absolute log2 fold changes for all proteins within that comparison.

### NAT quantification in plasma and bile

Plasma NATs were extracted in 300 μl acetonitrile with 10 μl of 1.25 μM C15-taurine (C15:0 NAT) in milliQ water as internal standard from 90 μl plasma. After 10 min shaking at 650 rpm at 25°C, extracts were centrifuged at 20,000 × g for 10 min with the temperature held at 20°C, and the supernatant was evaporated via speed vacuum. Bile was prepared by diluting 1 μl in 99 μl milliQ water containing 12.5 pmol C15:0 NAT. Plasma and bile samples were then analyzed as described (15).

### Bile acid quantification in plasma and bile

For plasma analysis, 10 μl of plasma extracted for NAT analysis was combined with 5 μl of 1 ppm of deuterated internal standards (TLCA-d4, TDCA-d4, DCA-d4, CA-d4, TCA-d4, TDCA-d4, LCA-d5, GCA-d4, GUDCA-d4, GCDCA-d4, GDCA-d4, and CDCA-d4) in methanol and 35 μl of LC/MS grade methanol. For bile analysis, 1.25 μl of bile prepared for NAT analysis were diluted in a total volume of 1 ml using milliQ water. 2.5 μl of this dilution was added to a vial containing 5 μl of 1 ppm deuterated internal standard (see above), 7.5 μl of LC/MS grade methanol, and 35 μl of milliQ water.

A standard curve was composed by combining 5 μl of standard (0.01, 0.05, 0.1, 0.5, 1 and 2 ppm of CA, CDCA, DCA, GCA, GCDCA, GDCA, GLCA, GUDCA, hyodeoxycholic acid [HDCA], LCA, MCA alpha, MCA beta, MCA gamma, MCA omega, muricholic acid, TCA, TCDCA, TDCA, tauro-HDCA [THDCA], TMCA alpha, TMCA beta, TMCA gamma, TMCA omega, TLCA, TUDCA, and UDCA) with 5 μl of 1 ppm of deuterated internal standards (see above), 10 μl milliQ water, and 30 μl of LC/MS grade methanol. An internal standard blank was created by replacing the volume of standard with LC/MS grade methanol.

Samples were placed in a prechilled autosampler held at 15°C and injected at 10 μl in a random order for low-resolution LC/MS analysis using an Agilent 1,290 Infinity II LC system coupled to an Agilent Jet Stream LC/MSD XT single quadrupole mass spectrometer. Analytes were separated over a Waters BEH C18 column (100 mm length × 2.1 mm internal diameter and 1.7 μm particle size) heated to 45°C using a gradient at a flow rate of 0.35 ml/min. Mobile phase A and mobile phase B were composed of 0.01% formic acid (LC-MS grade) in water (LC-MS grade) and 0.01% formic acid (LC-MS grade) in acetonitrile (LC-MS grade), respectively. The gradient used is included in Supplemental Table S1. Electrosprayed ions were detected in negative, single ion monitoring. The mass-to-charge ratios monitored and the accompanying MS parameters are included in Supplemental Table S2. The following parameters were used for the analysis: capillary voltage (V) = 4,000, source temperature (°C) = 350, sheath gas temperature (°C) = 400, gas flow (L/min) = 7, and sheath gas flow (L/min) = 12. Bile acids were quantified using the internal standard normalized curve. The retention times and internal standard used for quantitation are displayed in Supplemental Table S2. The limit of quantification was estimated as 20% lower than the lowest concentration included in the standard curve.

### NAT synthase activity assay

Mouse liver was pulverized and homogenized in 20 mM Hepes pH 7 with 50 mM NaCl. Protein was determined as described above, and NAT synthase activity was measured in 50 μg protein. The final reaction mixture contained 10 mM taurine and was initiated by adding 100 μM acyl-CoA (C22:6-CoA) in DMSO. After 5 min incubation, the reaction was stopped by the addition of four volumes of ice-cold methanol.

Afterward, samples were centrifuged at 20,000 × g for 10 min, and supernatants were used for qualitative analysis as described in (15).

### Hepatic lipid uptake

WT male mice were fed a high-fat/sugar/cholesterol diet (Research diets D9100310) for a total of 5 weeks and 10 days before the experiment siFatp5 (2 mg/kg, s.c.) was injected. On the experimental day, mice were fasted for four hours, followed by an oral gavage of 200 μl olive oil with ^3^H-triolein tracer (5 μCi). Blood was collected 1, 2, and 4 h post gavage, and tissues were collected after four hours. All collected tissues were weighed and digested in 1 M sodium hydroxide at 65°C for 1 h, and radioactivity was measured using a scintillation counter (Hidex 600 SLe). Plasma and bile radioactivity were measured in 10 or 1 μl, respectively.

### Statistics

All statistical analyses were performed using GraphPad Prism 10.5 unless otherwise specified. Data are presented as mean ± SEM. Statistically significant differences were determined by using two-tailed Student's *t* test, false discovery rate-corrected multiple t-tests, 1-wayANOVA followed by the Dunnett post-hoc test with vehicle as the control group, or two-way ANOVA followed by the Sidak post hoc analysis where appropriate. The following significance levels were used: ∗*P* ≤ 0.05, ∗∗*P* < 0.01, ∗∗∗*P* < 0.001, ∗∗∗∗*P* < 0.0001.

## Results

### Liver RNA-sequencing identifies a potential NAT synthesis gene

**Fig. 1:**
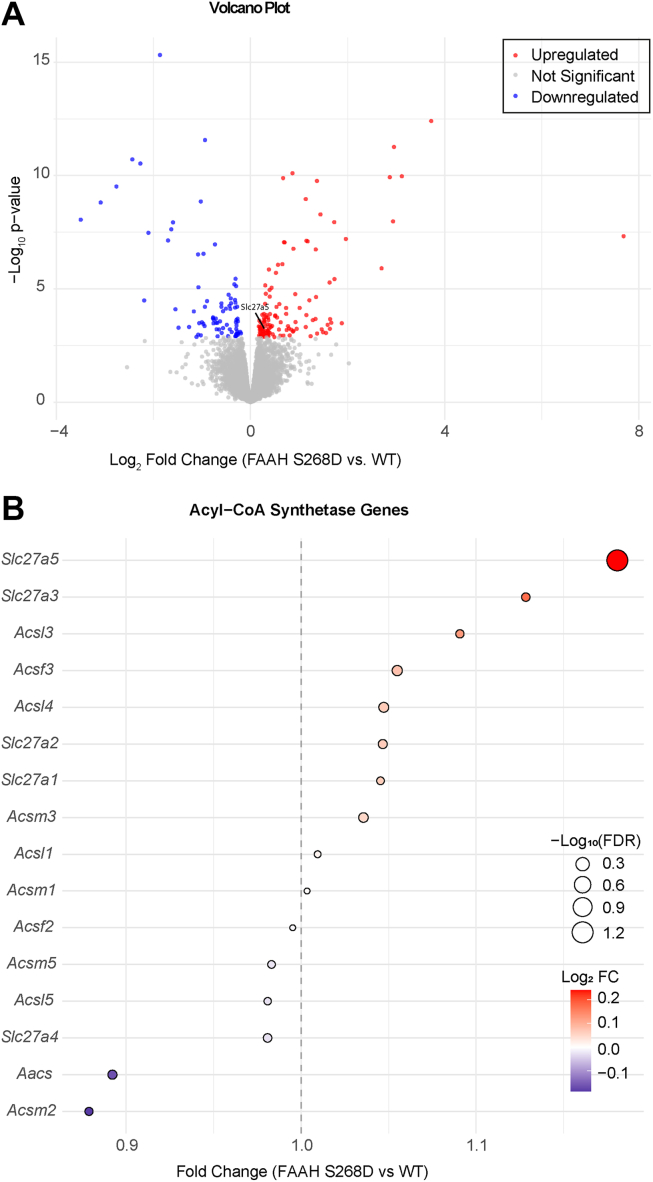
Liver RNA-sequencing identifies a potential NAT synthesis gene. Male WT and FAAH S268D mice were fed a HFD for 3 days, and RNA sequencing of the livers was performed. (A) Volcano plot of differentially expressed genes between WT and FAAH S268D mice (n = 6–10). (B) Bubble plot of all detected acyl-CoA synthetase genes. Genes were considered significant with an FDR < 0.1.

Due to the overlap in bile acid conjugation and NAT synthesis, we examined the effects of chronic elevation of NATs on hepatic fatty acid metabolism to identify additional overlap. We performed RNA sequencing in the livers of mice unable to hydrolyze NATs (FAAH S268D) (9) fed a high-fat diet for 3 days to increase fatty acid availability. In total, 204 genes were significantly changed between WT and FAAH S268D mice, of which 124 were upregulated and 80 were downregulated compared to WT (Fig. 1A). We specifically scrutinized genes related to bile acid or fatty acid metabolism with roles in fatty acid activation, as for hepatic NAT synthesis mediated by BAAT, an activated acyl-CoA is necessary to be conjugated to taurine (15). Out of all measured genes (Supplemental Table S3), we detected 16 acyl-CoA synthetase genes; however, only *Slc27a5* expression was significantly altered between the groups (Fig. 1B). *Slc27a5*, encoding the liver-specific protein FATP5, the ortholog of rat bile acid CoA ligase (BAL) (3), is a versatile protein, involved in hepatic fatty acid transport (20, 21), activating long- and very long-chain fatty acids by adding CoA (22), and the CoA ligation on bile acids in the liver (23, 24). As we have previously established that BAAT exerts a dual function in the liver by conjugating taurine to both bile acids and PUFAs (15), we hypothesized that FATP5 is directly involved in hepatic NAT synthesis by providing long-chain acyl-CoA upstream of BAAT.

### In vivo knockdown of FATP5 to investigate its role in NAT synthesis

**Fig. 2:**
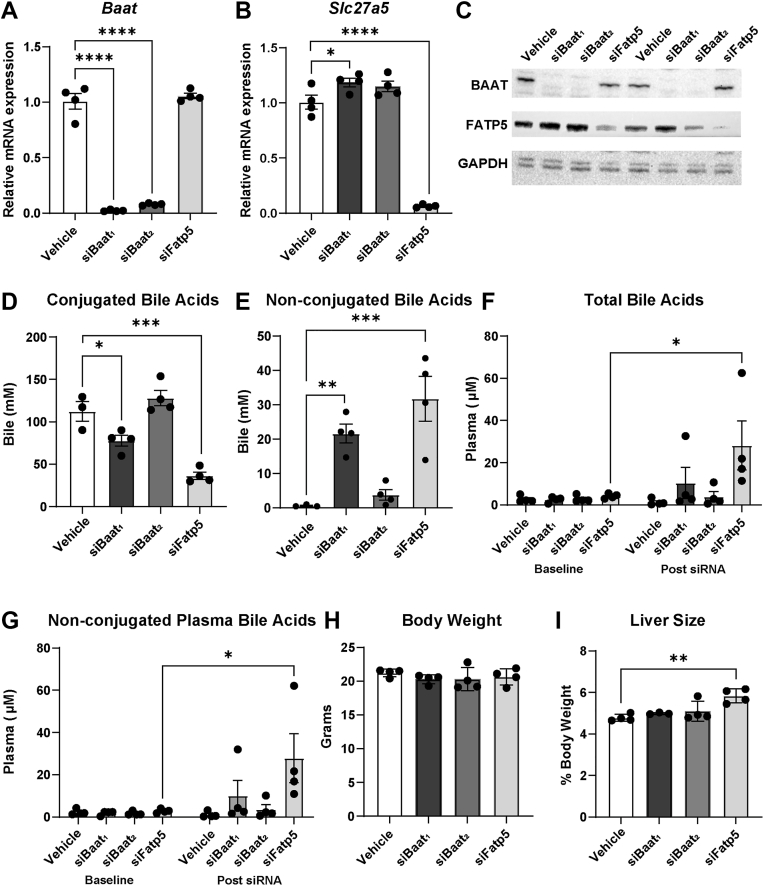
Successful in vivo silencing of the bile acid conjugation pathway. Female WT mice received either vehicle or siRNA (s.c., 10 mg/kg) two weeks prior to experiments. Hepatic gene expression of (A) *Baat* and (B) *Slc27a5*. (C) Hepatic protein expression by Western blot. Total biliary (D) conjugated and (E) non-conjugated bile acids. Plasma (F) total bile acids and (G) non-conjugated bile acids before and after siRNA administration. (H) End body weight. (I) Liver size as percent of body weight. n = 3–4. Data are represented as mean ± SEM and the following significance intervals are used: ∗*P* < 0.05, ∗∗*P* ≤ 0.01, ∗∗∗*P* ≤ 0.001, ∗∗∗∗*P* ≤ 0.0001.

**Supplemental Figure S1:**
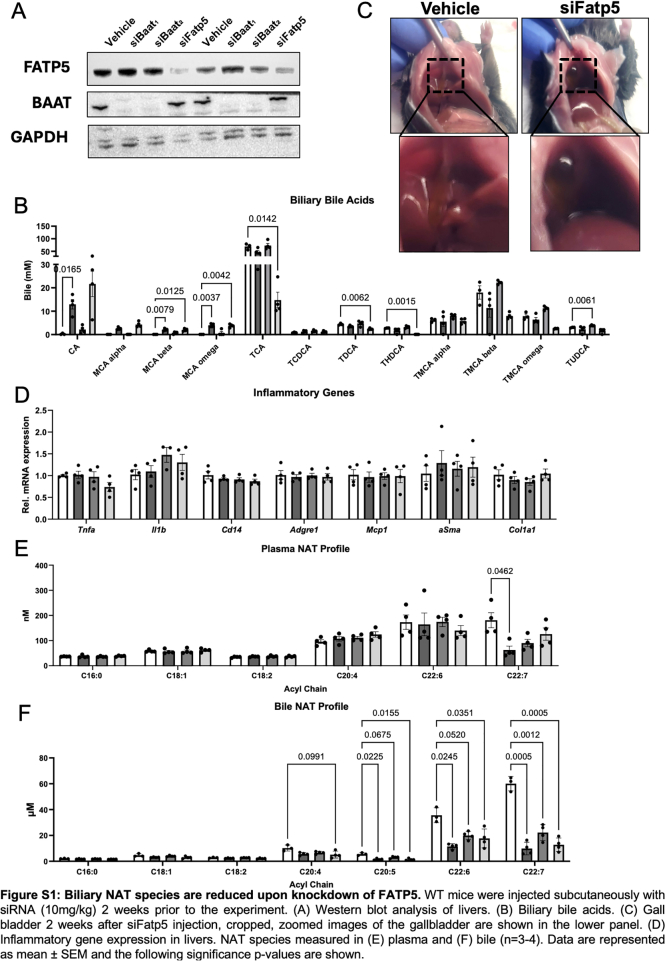


To determine if FATP5 is involved in hepatic NAT synthesis, we performed an siRNA-mediated knockdown of FATP5 and our established target, BAAT (15) in WT mice. Silencing of BAAT was used to validate the knockout model (15) and as a comparison to FATP5. A single dose of siRNA (10 mg/kg) against *Baat* (siBaat_1_ or siBaat_2_) or *Slc27a5* (siFatp5) resulted in a >90% reduction in *Baat* or *Slc27a5* mRNA expression (Fig. 2A, B). *Baat* knockdown slightly increased *Slc27a5* expression (Fig. 2B), whereas protein expression was unaltered (Fig. 2C, Supplemental Fig. S1A). To further validate our model, we measured bile acids in plasma and bile. siBaat_1_ exhibited the expected reduction of conjugated bile acids and elevation of unconjugated bile acids, whereas siBaat_2_ had little effect on these measures (Fig. 2D, E, Supplemental Fig. S1B). As anticipated from the *Fatp5*^*−/−*^ mouse (25), *Fatp5* knockdown reduced conjugated and elevated unconjugated bile acids in bile (Fig. 2D, E), thus validating the model. Unconjugated bile acids mediated the increase in plasma bile acids upon siFatp5 treatment (Fig. 2F, G). We did not observe a noticeable effect on body weight with knockdown of either protein (Fig. 2G); however, reduced FATP5 expression increased liver size (Fig. 1I) and increased gall bladder size (Supplemental Fig. S1C), as previously described (26). Neither lack of BAAT nor FATP5 had any impact on plasma lipid levels or liver inflammation and damage (Table 2, Supplemental Fig. S1D). Loss of FATP5 exerted a larger effect on bile acid conjugation than BAAT, potentially due to different turnover rates of the proteins or nonenzymatic synthesis of the conjugated bile acid when the bile acid-CoA is present in close proximity to taurine.

**Table 2:** Plasma parameters of WT mice treated with siRNA

	Vehicle	siBaat_1_	siBaat_2_	siFatp5
TG [mg/dL]	44.4 ± 4.1	40.9 ± 1.8	41.1 ± 3.5	43.0 ± 2.4
TC [mg/dL]	109.2 ± 7.9	113.2 ± 5.5	105.5 ± 10.5	105.4 ± 6.2
NEFA [mmol/L]	0.31 ± 0.04	0.31 ± 0.04	0.24 ± 0.03	0.29 ± 0.03
ALT [IU/L]	11.9 ± 1.6	10.8 ± 0.4	11.7 ± 0.6	12.5 ± 1.1

Data are presented as mean ± SEM (n = 4) and were analyzed with 1-way ANOVA followed by Dunnett post-hoc test, where vehicle is designated as the control group.

### PUFA-containing NATs and conjugated bile acids share hepatic pathways for synthesis

**Fig. 3:**
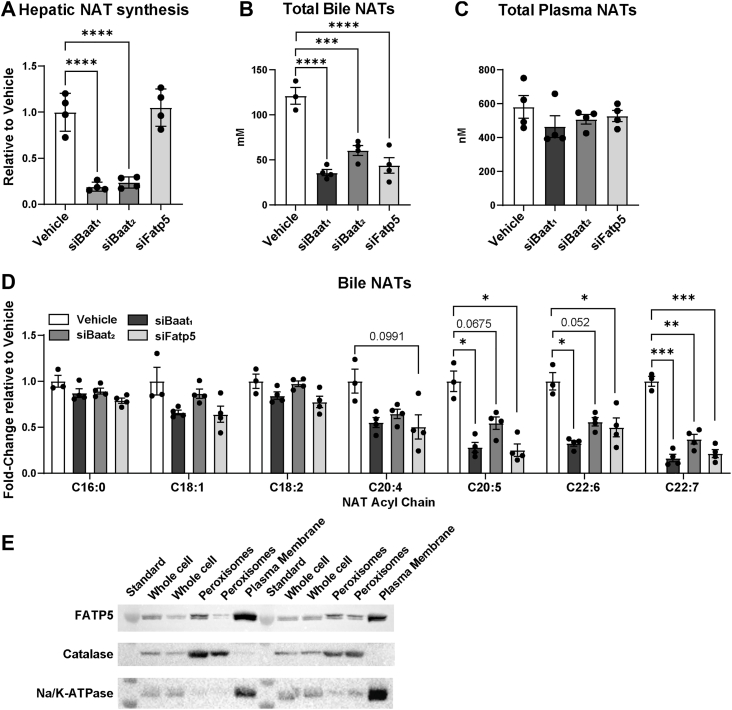
PUFA-NATs and bile acids share the hepatic conjugation pathway. Female WT mice received either vehicle or siRNA (s.c., 10 mg/kg) two weeks prior to experiments. (A) Hepatic NAT synthesis activity using C22:6-CoA as substrate with 50 μg liver protein (n = 4). Total NAT levels in (B) plasma and (C) bile (n = 3–4). (D) NAT profile of bile. (E) Western blot analysis of WT liver whole cell, peroxisome, or plasma membrane-enriched fractions (n = 4). Data are represented as mean ± SEM, and the following significance intervals are used: ∗*P* < 0.05, ∗∗*P* ≤ 0.01, ∗∗∗*P* ≤ 0.001, ∗∗∗∗*P* ≤ 0.0001.

After validating the models for effects on bile acids, we next set out to determine how the loss of FATP5 alters NAT synthesis and in vivo levels. Loss of BAAT reduced hepatic NAT synthase activity by approximately 80%, but loss of FATP5 did not affect this activity when the acyl-CoA is provided (Fig. 3A). A similar reduction in NAT synthase activity was observed in *Baat*^*−/−*^ livers (15). Importantly, the lack of FATP5 reduced biliary total NAT levels to the same extent as the loss of BAAT (Fig. 3B), indicating an essential role of FATP5 in the hepatic NAT synthesis pathway that is upstream of BAAT. Although biliary NAT levels were significantly reduced with silencing of BAAT or FATP5, plasma NAT levels remained unaltered (Fig. 3C, Supplemental Fig. S1E), indicating that the hepatic NAT synthesis provides NATs for secretion into bile, and plasma NAT levels are separately regulated. PUFA-containing NATs in bile were highly affected by impaired synthesis (Fig. 3D, Supplemental Fig. S1F), in line with previous observations that *Baat*^*−/−*^ mice had the greatest loss of PUFA-containing NATs (15). In line with the peroxisomal localization of BAAT, we observed localization of FATP5 in the peroxisome, along with the plasma membrane (Fig. 3E). The finding that FATP5 knockdown had similar effects to the lack of BAAT indicates that FATP5 may be partly responsible for the apparent preference for synthesizing these PUFA-containing NAT species in liver peroxisomes.

### FATP5 generates acyl-CoA directly for hepatic NAT synthesis

**Fig. 4:**
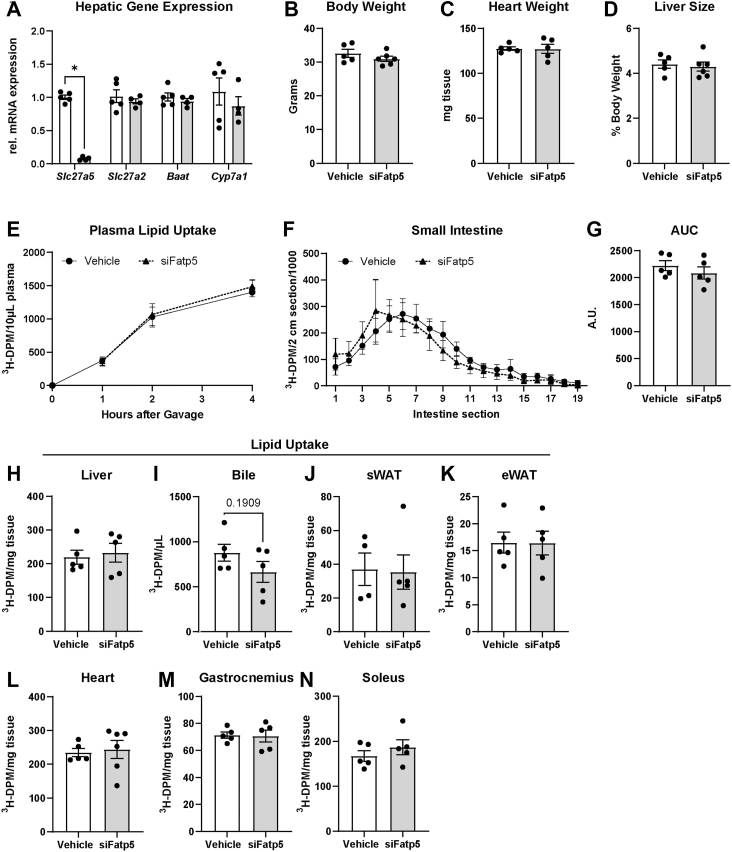
Low dose of siFatp5 does not alter liver size or lipid uptake. Male WT mice, fed a HFFC diet for a total of 5 weeks, received either vehicle or siRNA (s.c., 2 mg/kg) 10 days prior to experiments. (A) Hepatic mRNA expression of bile acid synthesis-related genes. (B) End body weight. (C) Heart weight. (D) Liver size. (E–N) Mice received a ^3^H-triolein oil gavage and tissues/fluids were collected 4h after. Lipid uptake in (E) plasma, (F, G) small intestine sections, (H) liver, (I) bile, (J) subcutaneous white adipose tissue (sWAT), (K) epidydimal WAT (eWAT), (L) heart, (M) gastrocnemius, and (N) soleus. n = 4–5. Data are represented as mean ± SEM, and the following significance intervals are used: ∗*P* < 0.05.

FATP5 has previously been implicated in hepatic fatty acid and bile acid metabolism (20, 21, 22, 23, 24, 25, 27), as well as the activation of hepatic stellate cells (26). To ensure the lack of biliary NATs was not due to general alterations in lipid metabolism, we reduced the siRNA dose 5-fold and still observed a significant reduction in *Slc27a5* gene expression without alterations in other bile acid synthesis genes or compensatory upregulation of *Scl27a2*, encoding for FATP2 (Fig. 4A). Importantly, body weight, as well as heart and liver size, remained unchanged with the lower siFatp5 dose (Fig. 4B–D), overcoming potential confounding factors seen in *Fatp5*^*−/−*^ mice (27). Moreover, at the lower siRNA dose, dietary fatty acid absorption and hepatic lipid uptake capacity after a ^3^H-labeled triolein oil bolus were unchanged and did not cause a redistribution of fatty acids into bile, adipose depots, or muscle (Fig. 4E–N), as reported for the *Fatp5*^*−/−*^ mouse (27). Thus, the reduction in apparent NAT synthesis was not due to a lack of dietary fatty acid absorption or hepatic uptake limiting the available substrate. Taken together, the lower dose enabled a targeted study of hepatic NAT and bile acid homeostasis without altering liver size or fatty acid distribution.

**Fig. 5:**
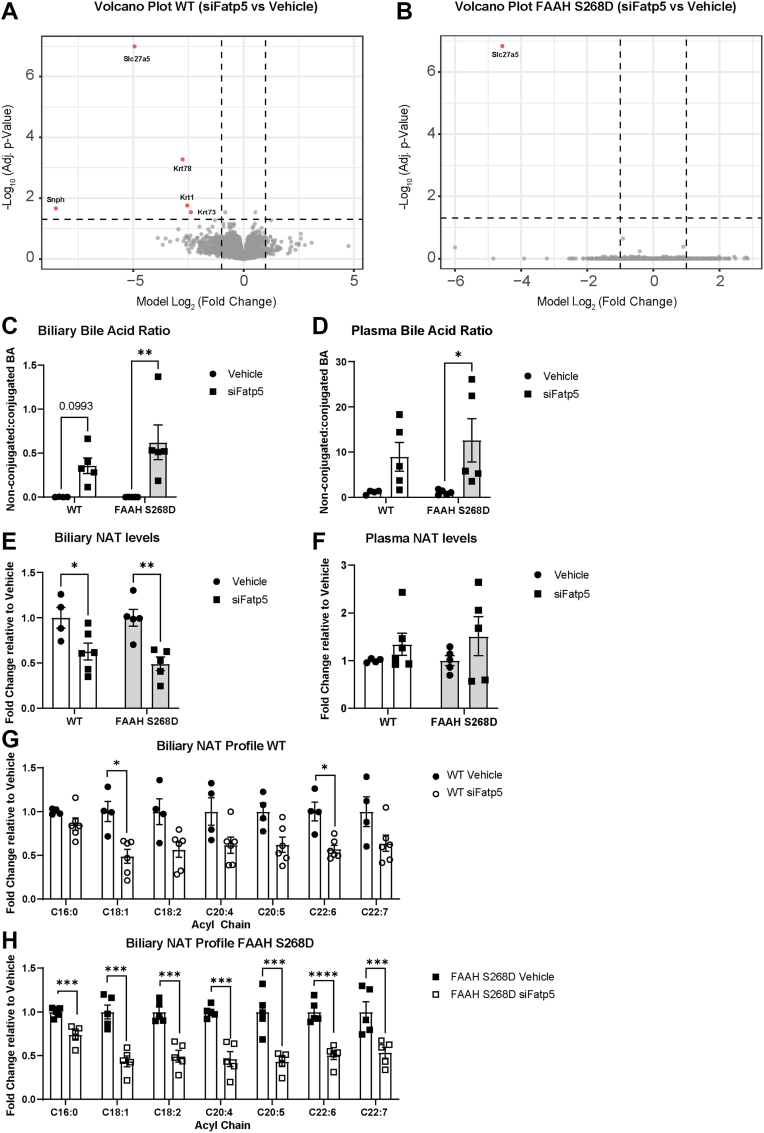
FATP5 is necessary for hepatic NAT synthesis. Male WT and littermate FAAH S268D mice, fed a HFFC diet for a total of 4 weeks, received either vehicle or siRNA (s.c., 2 mg/kg) two weeks prior to experiments. Volcano plot of liver proteomics analysis of (A) WT and (B) FAAH S268D mice (n = 4–5). Ratio of nonconjugated to conjugated bile acids in (C) bile or (D) plasma of WT (n = 4–6) and FAAH S268D mice (n = 5–6). (E) Total biliary NAT levels in WT (n = 4–6) and (H) FAAH S268D mice (n = 5–6). (F) Total plasma NAT concentrations in WT (n = 4–6) and FAAH S268D mice (n = 5–6). Biliary NAT profiles in (G) WT (n = 4–6) and (H) FAAH 268D mice (n = 5–6). Data are represented as mean ± SEM, and the following significance intervals are used: ∗*P* < 0.05, ∗∗*P* ≤ 0.001, ∗∗∗*P* ≤ 0.001, ∗∗∗∗*P* ≤ 0.0001.

To further evaluate the requirement of FATP5 in NAT synthesis, we utilized mice with impaired NAT hydrolysis, the FAAH S268D mouse (9), to eliminate altered NAT degradation as a potential confounding factor. Using proteomics, we confirmed the lack of FATP5 protein in both genotypes and observed very few other changes in the hepatic proteome (Fig. 5A, B), suggesting that the observed effects are due to the lack of FATP5. We observed a highly elevated ratio of non-conjugated to conjugated biliary and plasma bile acids in WT and FAAH S268D samples (Fig. 5C, D). Together with the unaltered expression of *Cyp7a1*, the rate-limiting step in bile acid synthesis (Fig. 4A), this suggests that the buildup of non-conjugated bile acids is due to a lack of FATP5 function. As observed with the higher dose (Fig. 2C, D), low-dose siFatp5 reduced total biliary NAT levels in FAAH S268D and littermate WT controls (Fig. 5E), while plasma NAT levels were not significantly altered (Fig. 5F). Importantly, in WT and even more pronounced in FAAH S268D mice, silencing of FATP5 with low-dose siFatp5 reduced all biliary long-chain fatty acyl-containing NAT species (Fig. 5G, H). Thus, FATP5 provides fatty acyl-CoA for hepatic long-chain NAT synthesis and is involved in NAT synthesis upstream of BAAT.

## Discussion

NATs are endogenous, bioactive lipid metabolites (9), which have been evolutionarily conserved from the intestinal juice of invertebrates (5, 6, 7, 28) to the bile of mammals (10). Although the NAT degradation pathway has been known for the past two decades, the in vivo synthesis pathways have only recently been investigated. BAAT, previously well-described to perform bile acid amidation (3, 4), exerts a dual function by also conjugating hepatic polyunsaturated acyl-CoA to taurine (15). In addition, ACNATs have been shown to have NAT synthase activity in vitro (29), but their in vivo role remains to be elucidated. Additional enzymes involved in the hepatic NAT production pathway were unknown. Here, we identified FATP5 as the hepatic NAT acyl-CoA synthetase using transcriptomics and siRNA-mediated knockdown.

Pharmacological inhibition of NAT degradation by FAAH leads to a rapid accumulation of NATs in the liver (8, 12), suggesting an active pathway for hepatic NAT production. Due to similar chemical properties of PUFA-containing NATs and steroidal conjugated bile acids and their shared conjugation enzyme, BAAT, we hypothesized that upstream enzymes might also be shared with those that ligate CoA onto the bile acid. BAAT-mediated NAT synthesis requires the fatty acid to be activated with a CoA before conjugation to taurine (15), suggesting the liver expresses an acyl-CoA synthetase with a previously unknown function in directing acyl-CoAs to NAT synthesis. Therefore, we specifically examined genes involved in bile and fatty acid metabolism, with distinct roles in fatty acid activation, and identified and confirmed FATP5 (encoded by *Slc27a5*) as an acyl-CoA synthetase prior to taurine conjugation via BAAT in hepatic NAT synthesis. By using siRNA-mediated in vivo knockdown, including concentration modulation, we overcame apparent alterations in hepatic and systemic lipid homeostasis and hepatomegaly observed in the *Fatp5*^*−/−*^ mouse (27). In this way, we could study and confirm its role specifically in hepatic NAT production.

The development of digestive juice across the animal kingdom seems to have transitioned from fatty acid-based detergents in invertebrates (5, 30) to primarily bile alcohols and/or bile acids in vertebrates (31). Accordingly, NATs, but not bile acids, are present in invertebrate aquatic animals (5, 6, 7) and may represent an early form of conjugated bile acids in species lacking sterol synthesis (32), suggesting a preserved evolutionary detergent remnant. The bile acid conjugation machinery, BAAT and FATP5, is present only in vertebrates (33, 34), indicating other enzyme(s) could be involved in invertebrate fatty acid-amino acid conjugation. By contrast, the NAT degradation enzyme FAAH is a highly conserved protein across the eukaryotic lineage (35), including plant homologs (36, 37).

FATP5, known under various synonyms (very long-chain acyl-CoA synthetase - VLACSR, Solute carrier family 27 member 5 - SLC27A5, BAL), also exerts a variety of biological functions involved in hepatic fatty acid and bile acid metabolism (20, 21, 22, 23, 24, 25, 27), as well as hepatic stellate cell activation (26) and tumorigenesis (38, 39). Mice lacking FATP5 exhibit hepatomegaly, systemic fatty acid redistribution from the liver to peripheral organs (27), and a higher proportion of non-conjugated bile acids (25, 40), and tend to develop fibrosis (26). The latter finding of increased fibrosis has been reported in patients with defects in FATP5 (41, 42). Recently, reduced FATP5 expression has been associated with liver fibrosis, cirrhosis, and hepatocellular carcinoma in humans (26, 38, 39, 43, 44), and mouse studies confirmed FATP5's protective role for fibrosis, liver cancer development, and metastasis (26, 38, 39). Due to the multiple hepatic pathways in which FATP5 appears to be involved, we needed to modulate the siRNA-mediated knockdown to a level that allowed us to confirm its role in NAT synthesis without potential confounding disturbances to hepatic and whole-body metabolism. Other acyl-CoA synthetases might contribute to hepatic NAT production by providing an acyl-CoA thioester; however, none except FATP5 (23, 24) and FATP2 (23) have been described in bile acid metabolism. FATP2 synthesizes C27-bile acid-CoA thioesters but does not activate primary or secondary C24 bile acids (23), and liver long-chain acyl-CoA synthase activity can be compensated upon loss of FATP2 (45), whereas BAL activity mediated by FATP5 is nonredundant (25, 46). siRNA-mediated FATP5 depletion did not result in compensatory regulation of hepatic FAPT2, in line with previous observations in the *Fatp5*^*−/−*^ mouse or virus-mediated FATP5-knockdown (27, 47, 48), which both resulted in a bile acid conjugation defect upon loss of FATP5 (25) but not hepatic FATP2 (46). Given hepatic compensation of long-chain acyl-CoA synthase activity upon FATP2 knockdown, it would be hard to specifically assess its role in NAT synthesis, as we would expect FATP5 to compensate in hepatic NAT synthesis. Mechanistically, it seems that whole body *Fatp2*^*−/−*^ affects more fatty acid-related pathways, specifically the long-chain fatty acid profile and degradation products (lipoxins and prostaglandins), than bile acid-related pathways in the liver (49). If hepatic FAPT2 plays a role in the synthesis of biliary NATs, this role is likely to be minor. Both FATP5 and BAAT depletion produce a similar reduction in biliary NAT levels, suggesting only a marginal contribution from other acyl-CoA synthetases. However, whether and to what extent FATP2 participates in hepatic NAT synthesis remains elusive and should be addressed in future studies. Recently, acute hepatic, virus-mediated *Acsl1* knockdown has been shown to reduce bile acid synthesis by cholesterol shunting and transcriptional control (50), but ACSL1 is not directly involved in bile acid-CoA formation (23). Taken together, other acyl-CoA synthetases might have an additional role in providing activated fatty acids for hepatic and extra-hepatic NAT production, but FATP5 seems to be necessary for PUFA-containing NAT synthesis in the liver.

Similar to previous observations that *Baat*^*−/−*^ affects biliary NAT levels (15), siRNA-mediated knockdown of BAAT or FATP5 lowered biliary NATs without significantly altering plasma NAT levels, suggesting that plasma NATs derive from a nonhepatic source (12, 15). The kidney also displays high NAT synthesis activity (12, 15), making it a good candidate for supplying plasma NATs. Both FATP5 and BAAT are liver-specific enzymes, suggesting that other enzymes/pathways are responsible for this activity in the kidney and warrant further investigation.

Depleting FATP5 had a greater effect on bile acid conjugation compared to depleting BAAT. This effect is also evident in the corresponding knockout mice (25, 51). FATP5 activates bile acids for conjugation prior to BAAT amidation, which can be performed by ACNAT1 and, potentially, ACNAT2 (29, 33, 51), resulting in unusual bile acids in the *Baat*^*−/−*^ mouse (51, 52). In humans, however, *BAAT* mutations lead to severe malabsorption and a complete absence of conjugated bile acids (41), likely due to a lack of compensatory enzymes, such as ACNATs, which are not expressed in humans (33). In contrast, a patient carrying a mutation in the *SLC27A5* gene was described as retaining a small proportion of conjugated bile acids (42), suggesting compensation by other acyl-CoA synthetases occurs in this extreme condition. Together, in mice, it appears that FATP5 generates all substrates for downstream bile acid conjugation in the liver, whereas BAAT loss may be partially compensated for.

Due to lipid malabsorption as well as altered bile acid synthesis and signaling in the *Baat*^*−/−*^ mouse (51, 52), it is complex to use this model to study the necessity of liver-derived NATs. The use of siRNA-mediated knockdown in WT mice avoids these complications, making it useful for future studies to disentangle altered hepatic NAT production dependent or independent of altered bile acid profiles.

Amphipathic molecules, such as conjugated bile acids, act as detergents, promoting lipid emulsification through micelle formation. Similarly, PUFA-containing NATs structurally follow the same pattern with their lipophilic acyl chain amidated to hydrophilic taurine to increase water solubility and form micelles (10). Given the shared structural and physicochemical properties of bile acids and PUFA-containing NATs, it is not surprising that the same enzymes synthesize these metabolites. In addition to the taurine-conjugating enzyme BAAT, which has a dual role in NAT and bile acid conjugation, we identified another hepatic enzyme, FATP5, as having roles in both pathways. Moreover, because these enzymes have dual roles, care should be taken when targeting either BAAT or FATP5 to alter bile acid levels, as this may also affect NAT metabolism.

Intracellularly, bile acids and PUFA-containing NATs are conjugated by BAAT in the peroxisomes (15). FATP5 localization was confirmed on the basal plasma membrane facing the space of Disse (27) supporting its role in hepatic lipid uptake and fatty acid retention, however, acyl-CoA synthase activity of very long chain fatty acids (>22 carbons) in the rat liver was localized to the peroxisome (53). We have further confirmed that FATP5 is also expressed in liver peroxisomes, supporting the idea of peroxisomal NAT synthesis in the liver.

Taken together, our study identified FATP5 as an acyl-CoA synthetase that directs fatty acids to hepatic NAT synthesis as a two-step metabolic pathway. Moreover, this work positions NATs more similarly to bile acids, not only chemically but also by sharing the hepatic conjugation enzymes, and provides insights into the hepatic NAT production pathway.

## Data Availability

Proteomics data have been deposited at the ProteomeXchange Consortium via the PRIDE partner repository (54) with the dataset identifier PXD070306. RNAseq data have been deposited in ArrayExpress with the accession number E-MTAB-15752: https://www.ebi.ac.uk/biostudies/ArrayExpress/studies/E-MTAB-15752?key=0fd51732-8813-4062-a20c-45c35492e4bc. All deposited data are publicly available as of the date of publication. Data will be shared upon reasonable request to the corresponding author, Trisha J. Grevengoed, University of Copenhagen, grevengoed@sund.ku.dk.

## Supplemental Data

This article contains supplemental data.

## Declaration of Interest

B. P. G. is an employee and shareholder of Alnylam Pharmaceuticals. All other authors declare no competing interests.

## References

[1] Fleishman J.S., Kumar S. (2024). Bile acid metabolism and signaling in health and disease: molecular mechanisms and therapeutic targets. Sig. Transduct. Target. Ther..

[2] Rembacz K.P., Woudenberg J., Hoekstra M., Jonkers E.Z., van den Heuvel F.A.J., Buist-Homan M. (2010). Unconjugated bile salts shuttle through hepatocyte peroxisomes for taurine conjugation. Hepatology.

[3] Falany C.N., Xie X., Wheeler J.B., Wang J., Smith M., He D. (2002). Molecular cloning and expression of rat liver bile acid CoA ligase. J. Lipid Res..

[4] Killenberg P.G., Jordan J.T. (1978). Purification and characterization of bile acid-CoA:amino acid N-acyltransferase from rat liver. J. Biol. Chem..

[5] Holwerda D.A., Vonk H.J. (1973). Emulsifiers in the intestinal juice of crustacea. Isolation and nature of surface-active substances from *Astacus leptodactylus* Esch. and *Homarus vulgaris* L. Comp. Biochem. Physiol. B Comp. Biochem..

[6] Huang R., Peng Y., Zhou X., Yang X., Liu Y. (2013). A new taurine derivative from South China Sea marine sponge Axinella sp. Nat. Product. Res..

[7] Zhou X., Xu T., Wen K., Yang X.-W., Xu S.-H., Liu Y. (2010). New N-Acyl taurine from the sea urchin Glyptocidaris crenularis. Biosci. Biotechnol. Biochem..

[8] Long J.Z., LaCava M., Jin X., Cravatt B.F. (2011). An anatomical and temporal portrait of physiological substrates for fatty acid amide hydrolase. J. Lipid Res..

[9] Grevengoed T.J., Trammell S.A.J., McKinney M.K., Petersen N., Cardone R.L., Svenningsen J.S. (2019). N-acyl taurines are endogenous lipid messengers that improve glucose homeostasis. Proc. Natl. Acad. Sci. U. S. A..

[10] Grevengoed T.J., Trammell S.A., Svenningsen J.S., Makarov M.V., Nielsen T.S., Jacobsen J.C.B. (2021). An abundant biliary metabolite derived from dietary omega-3 polyunsaturated fatty acids regulates triglycerides. J. Clin. Invest..

[11] Saghatelian A., Cravatt B.F. (2005). Discovery metabolite profiling — forging functional connections between the proteome and metabolome. Life Sci..

[12] Saghatelian A., McKinney M.K., Bandell M., Patapoutian A., Cravatt B.F. (2006). A FAAH-regulated class of N-acyl taurines that activates TRP ion channels. Biochemistry.

[13] Sasso O., Pontis S., Armirotti A., Cardinali G., Kovacs D., Migliore M. (2016). Endogenous N-acyl taurines regulate skin wound healing. Proc. Natl. Acad. Sci. U. S. A..

[14] Saghatelian A., Trauger S.A., Want E.J., Hawkins E.G., Siuzdak G., Cravatt B.F. (2004). Assignment of endogenous substrates to enzymes by global metabolite profiling. Biochemistry.

[15] Trammell S.A.J., Gamon L.F., Gotfryd K., Michler K.T., Alrehaili B.D., Rix I. (2023). Identification of bile acid-CoA:amino acid N-acyltransferase as the hepatic N-acyl taurine synthase for polyunsaturated fatty acids. J. Lipid Res..

[16] Liao Y., Smyth G.K., Shi W. (2013). The subread aligner: fast, accurate and scalable read mapping by seed-and-vote. Nucleic Acids Res.

[17] Frankish A., Diekhans M., Ferreira A.-M., Johnson R., Jungreis I., Loveland J. (2019). GENCODE reference annotation for the human and mouse genomes. Nucleic Acids Res.

[18] Liao Y., Smyth G.K., Shi W. (2014). featureCounts: an efficient general purpose program for assigning sequence reads to genomic features. Bioinformatics.

[19] Robinson M.D., McCarthy D.J., Smyth G.K. (2010). edgeR : a bioconductor package for differential expression analysis of digital gene expression data. Bioinformatics.

[20] Berger J., Truppe C., Neumann H., Forss-Petter S. (1998). A novel relative of the very-long-chain Acyl-CoA synthetase and fatty acid transporter protein genes with a distinct expression pattern. Biochem. Biophysical Res. Commun..

[21] Hirsch D., Stahl A., Lodish H.F. (1998). A family of fatty acid transporters conserved from mycobacterium to man. Proc. Natl. Acad. Sci. U. S. A..

[22] Steinberg S.J., Kemp S., Braiterman L.T., Watkins P.A. (1999). Role of very-long-chain acyl–coenzyme A synthetase in X-linked adrenoleukodystrophy. Ann. Neurol..

[23] Mihalik S.J., Steinberg S.J., Pei Z., Park J., Kim D.G., Heinzer A.K. (2002). Participation of two members of the very long-chain Acyl-CoA synthetase family in bile acid synthesis and recycling. J. Biol. Chem..

[24] Steinberg S.J., Mihalik S.J., Kim D.G., Cuebas D.A., Watkins P.A. (2000). The human liver-specific homolog of very long-chain Acyl-CoA synthetase is Cholate:CoA ligase. J. Biol. Chem..

[25] Hubbard B., Doege H., Punreddy S., Wu H., Huang X., Kaushik V.K. (2006). Mice deleted for fatty acid transport protein 5 have defective bile acid conjugation and are protected from obesity. Gastroenterology.

[26] Wu K., Liu Y., Xia J., Liu J., Wang K., Liang H. (2023). Loss of SLC27A5 activates hepatic stellate cells and promotes liver fibrosis via unconjugated cholic acid. Adv Sci (Weinh).

[27] Doege H., Baillie R.A., Ortegon A.M., Tsang B., Wu Q., Punreddy S. (2006). Targeted deletion of FATP5 reveals multiple functions in liver metabolism: alterations in hepatic lipid homeostasis. Gastroenterology.

[28] Lester R., Carey M.C., Little J.M., Cooperatein L.A., Dowd S.R. (1975). Crustacean intestinal detergent promotes sterol solubilization. Science.

[29] Reilly S.-J., O'Shea E.M., Andersson U., O'Byrne J., Alexson S.E.H., Hunt M.C. (2007). A peroxisomal acyltransferase in mouse identifies a novel pathway for taurine conjugation of fatty acids. FASEB J..

[30] Vonk H.J. (1969). The properties of some emulsifiers in the digestive fluids of invertebrates. Comp. Biochem. Physiol..

[31] Hofmann A.F., Hagey L.R., Krasowski M.D. (2010). Bile salts of vertebrates: structural variation and possible evolutionary significance. J. Lipid Res..

[32] Kanazawa A. (2001). Sterols in marine invertebrates. Fisheries Sci..

[33] Kirilenko B.M., Hagey L.R., Barnes S., Falany C.N., Hiller M. (2019). Evolutionary analysis of bile acid-conjugating enzymes reveals a complex duplication and reciprocal loss history. Genome Biol. Evol..

[34] Watkins P.A., Maiguel D., Jia Z., Pevsner J. (2007). Evidence for 26 distinct acyl-coenzyme A synthetase genes in the human genome. J. Lipid Res..

[35] McKinney M.K., Cravatt B.F. (2005). Structure and function of fatty acid amide hydrolase. Annu Rev Biochem.

[36] Shrestha R., Dixon R.A., Chapman K.D. (2003). Molecular identification of a functional homologue of the mammalian fatty acid amide hydrolase *in Arabidopsis thaliana*. J. Biol. Chem..

[37] Haq I., Kilaru A. (2020). An endocannabinoid catabolic enzyme FAAH and its paralogs in an early land plant reveal evolutionary and functional relationship with eukaryotic orthologs. Sci Rep..

[38] Wang M.-D., Wang N.-Y., Zhang H.-L., Sun L.-Y., Xu Q.-R., Liang L. (2021). Fatty acid transport protein-5 (FATP5) deficiency enhances hepatocellular carcinoma progression and metastasis by reprogramming cellular energy metabolism and regulating the AMPK-mTOR signaling pathway. Oncogenesis.

[39] Gao Q., Zhang G., Zheng Y., Yang Y., Chen C., Xia J. (2020). SLC27A5 deficiency activates NRF2/TXNRD1 pathway by increased lipid peroxidation in HCC. Cell Death Differ..

[40] Ason B., Castro-Perez J., Tep S., Stefanni A., Tadin-Strapps M., Roddy T. (2011). ApoB siRNA-induced liver steatosis is resistant to clearance by the loss of fatty acid transport protein 5 (Fatp5). Lipids.

[41] Setchell K.D.R., Heubi J.E., Shah S., Lavine J.E., Suskind D., Al-Edreesi M. (2013). Genetic defects in bile acid conjugation cause fat-soluble vitamin deficiency. Gastroenterology.

[42] Chong C.P.K., Mills P.B., McClean P., Gissen P., Bruce C., Stahlschmidt J. (2012). Bile acid-CoA ligase deficiency—a new inborn error of bile acid metabolism. J. Inherit. Metab. Dis..

[43] Enooku K., Tsutsumi T., Kondo M., Fujiwara N., Sasako T., Shibahara J. (2020). Hepatic FATP5 expression is associated with histological progression and loss of hepatic fat in NAFLD patients. J Gastroenterol.

[44] Zhu L., Baker S.S., Liu W., Tao M.-H., Patel R., Nowak N.J. (2011). Lipid in the livers of adolescents with nonalcoholic steatohepatitis: combined effects of pathways on steatosis. Metabolism.

[45] Falcon A., Doege H., Fluitt A., Tsang B., Watson N., Kay M.A. (2010). FATP2 is a hepatic fatty acid transporter and peroxisomal very long-chain acyl-CoA synthetase. Am. J. Physiol. Endocrinol. Metab..

[46] Tharp K.M., Khalifeh-Soltani A., Park H.M., Yurek D.A., Falcon A., Wong L. (2016). Prevention of gallbladder hypomotility via FATP2 inhibition protects from lithogenic diet-induced cholelithiasis. Am. J. Physiol. Gastrointest. Liver Physiol..

[47] Doege H., Grimm D., Falcon A., Tsang B., Storm T.A., Xu H. (2008). Silencing of hepatic fatty acid transporter protein 5 in vivo reverses diet-induced non-alcoholic fatty liver disease and improves hyperglycemia. J. Biol. Chem..

[48] Uchiyama A., Aoyama T., Kamijo K., Uchida Y., Kondo N., Orii T. (1996). Molecular cloning of cDNA encoding rat very long-chain Acyl-CoA synthetase. J. Biol. Chem..

[49] Perez V.M., Gabell J., Behrens M., Wase N., DiRusso C.C., Black P.N. (2020). Deletion of fatty acid transport protein 2 (FATP2) in the mouse liver changes the metabolic landscape by increasing the expression of PPARα-regulated genes. J Biol Chem.

[50] Singh A.B., Dong B., Xu Y., Zhang Y., Liu J. (2019). Identification of a novel function of hepatic long-chain acyl-CoA synthetase-1 (ACSL1) in bile acid synthesis and its regulation by bile acid-activated farnesoid X receptor. Biochim. Biophys. Acta Mol. Cell Biol. Lipids.

[51] Neugebauer K.A., Okros M., Guzior D.V., Feiner J., Chargo N.J., Rzepka M. (2022). Baat gene knockout alters post-natal development, the gut microbiome, and reveals unusual bile acids in mice. J. Lipid Res..

[52] Alrehaili B.D., Lee M., Takahashi S., Novak R., Rimal B., Boehme S. (2022). Bile acid conjugation deficiency causes hypercholanemia, hyperphagia, islet dysfunction, and gut dysbiosis in mice. Hepatol. Commun..

[53] Uchida Y., Kondo N., Orii T., Hashimoto T. (1996). Purification and properties of rat liver peroxisomal very-long-chain Acyl-CoA synthetase. J. Biochem..

[54] Perez-Riverol Y., Bai J., Bandla C., García-Seisdedos D., Hewapathirana S., Kamatchinathan S. (2022). The PRIDE database resources in 2022: a hub for mass spectrometry-based proteomics evidences. Nucleic Acids Res..

