# Supplementary material for: The bile acid-CoA ligase, FATP5, is necessary for the synthesis of N-acyl taurines in the liver
Source: J Lipid Res. 2026 Mar 2;67(4):101012. doi: 10.1016/j.jlr.2026.101012 (PMC13050040; doi:10.1016/j.jlr.2026.101012)
Supplement: Supplemental Tables S1 and S2 [file mmc1.docx]

| RT (min) | %B | Flow Rate (mL/min) |
| --- | --- | --- |
| 0 | 25 | 0.35 |
| 2 | 25.32 | 0.35 |
| 4 | 25.71 | 0.35 |
| 6 | 26.44 | 0.35 |
| 8 | 27.76 | 0.35 |
| 9.99 | 27.76 | 0.35 |
| 10 | 28 | 0.35 |
| 12 | 28.1 | 0.35 |
| 14 | 32.59 | 0.35 |
| 16 | 38.59 | 0.35 |
| 18 | 46.3 | 0.35 |
| 20 | 55.94 | 0.35 |
| 26 | 75 | 0.35 |
| 26.01 | 100 | 0.35 |
| 28.1 | 100 | 0.35 |
| 28.2 | 20 | 0.35 |
| 30 | 20 | 0.35 |

Table S1. LC gradient.

| Bile Acid Name | Mass-to-Charge Ratio | RT (min) | Fragmentor (V) | Gain | IS |
| --- | --- | --- | --- | --- | --- |
| TMCA omega | 514.5 | 3.625 | 135 | 1 | TCA-d4 |
| TMCA alpha | 514.5 | 4.001 | 135 | 1 | TCA-d4 |
| TMCA beta | 514.5 | 4.228 | 135 | 1 | TCA-d4 |
| TMCA gamma | 514.5 | 6.895 | 135 | 1 | TCA-d4 |
| TUDCA | 498.6 | 9.22 | 135 | 1 | TCDCA-d4 |
| THDCA | 498.6 | 9.637 | 135 | 1 | TCDCA-d4 |
| TCA-d4 | 518.6 | 10.602 | 135 | 1 | N/A |
| TCA | 514.5 | 10.638 | 135 | 1 | TCA-d4 |
| GUDCA-d4 | 452.6 | 14.338 | 135 | 1 | N/A |
| MCA omega | 407.5 | 14.393 | 135 | 1 | CA-d4 |
| GUDCA | 448.5 | 14.406 | 135 | 1 | GUDCA-d4 |
| GCA-d4 | 468.6 | 15.146 | 135 | 1 | N/A |
| MCA alpha | 407.5 | 15.171 | 135 | 1 | CA-d4 |
| GCA | 464.6 | 15.177 | 135 | 1 | GCA-d4 |
| MCA beta | 407.5 | 15.809 | 135 | 1 | CA-d4 |
| TCDCA-d4 | 502.5 | 16.812 | 135 | 1 | N/A |
| TCDCA | 498.6 | 16.831 | 135 | 1 | TCDCA-d4 |
| MCA gamma | 407.5 | 17.32 | 135 | 1 | CA-d4 |
| Murocholic acid | 391.5 | 17.375 | 135 | 1 | UDCA-d4 |
| TDCA | 498.6 | 17.487 | 135 | 1 | TCDCA-d4 |
| CA-d4 | 411.6 | 18.224 | 135 | 1 | N/A |
| CA | 407.5 | 18.238 | 135 | 1 | CA-d4 |
| UDCA-d4 | 395.5 | 18.373 | 135 | 1 | N/A |
| UDCA | 391.5 | 18.401 | 135 | 1 | UDCA-d4 |
| GCDCA-d4 | 452.6 | 18.602 | 135 | 1 | N/A |
| GCDCA | 448.5 | 18.619 | 135 | 1 | GCDCA-d4 |
| HDCA | 391.5 | 18.628 | 135 | 1 | UDCA-d4 |
| GDCA-d4 | 452.6 | 19.05 | 135 | 1 | N/A |
| GDCA | 448.5 | 19.067 | 135 | 1 | GDCA-d4 |
| TLCA-d4 | 486.6 | 19.912 | 135 | 1 | N/A |
| TLCA | 482.5 | 19.931 | 135 | 1 | TLCA-d4 |
| CDCA-d4 | 395.5 | 20.791 | 135 | 1 | N/A |
| CDCA | 391.5 | 20.808 | 135 | 1 | CDCA-d4 |
| DCA-d4 | 395.5 | 21.092 | 135 | 1 | N/A |
| DCA | 391.5 | 21.109 | 135 | 1 | DCA-d4 |
| GLCA | 432.5 | 21.39 | 135 | 1 | TCDCA-d4 |
| LCA-d5 | 380.5 | 23.858 | 135 | 1 | N/A |
| LCA | 375.5 | 23.907 | 135 | 1 | LCA-d5 |

Table S2. Single ion monitoring parameters and internal standards used for quantitation.
